# The *CsMYB123* and *CsbHLH111* are involved in drought stress-induced anthocyanin biosynthesis in *Chaenomeles speciosa*

**DOI:** 10.1186/s43897-023-00071-2

**Published:** 2023-11-22

**Authors:** Yanshen Ren, Shuangyu Zhang, Qianyi Zhao, Yang Wu, Houhua Li

**Affiliations:** College of Landscape Architecture and Art, Northwest A&F University, Yangling, 712100 Shaanxi China

**Keywords:** *Chaenomeles speciosa*, Drought, *CsbHLH111*, *CsMYB123*, Anthocyanin

## Abstract

Drought stress has been demonstrated to enhance the biosynthesis of anthocyanins in the leaves, resulting in an increased aesthetic appeal. However, the molecular mechanisms underlying drought-induced anthocyanin biosynthesis in *Chaenomeles speciosa* remain unclear. In this study, the metabolites of *C. speciosa* leaves were analyzed, and it was found that the content of cyanidin-3-*O*-rutinoside increased significantly under drought stress. The differentially expressed genes *CsMYB123* and *CsbHLH111* were isolated by transcriptomics data analysis and gene cloning, and gene overexpression and VIGS experiments verified that both play important roles in anthocyanin biosynthesis. Subsequently, Y1H and Dual-luciferase reporter assay showed that *CsMYB123* binds to the promoters of anthocyanin biosynthesis-related structural genes (such as *CsCHI*, *CsF3H*, and *CsANS*), while *CsbHLH111* was shown to bind to the promoter of *CsCHI*, positively regulating its activity. Furthermore, BIFC and Y2H assays unveiled potential protein–protein interactions between CsMYB123 and CsbHLH111 at the cell nucleus. Collectively, these results shed light on the critical roles played by *CsMYB123* and *CsbHLH111* in anthocyanin biosynthesis, thus providing a valuable insight into understanding the molecular mechanisms of how the MYB and bHLH genes regulate anthocyanin biosynthesis in the process of leaf coloration in *C. speciosa*.

**Graphical Abstract:**

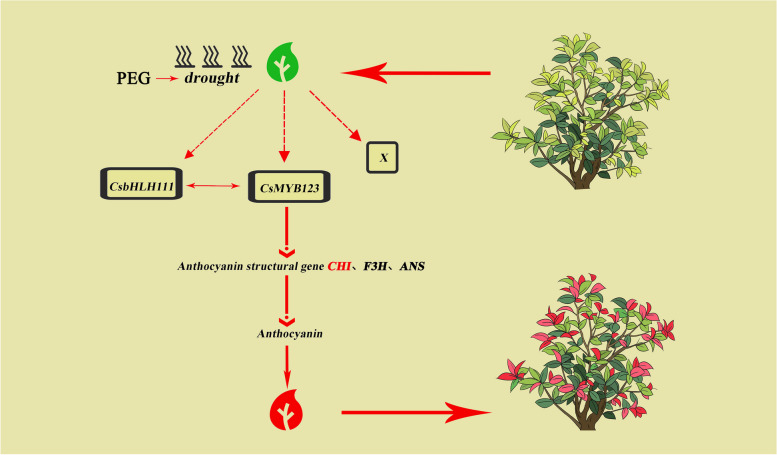

**Supplementary Information:**

The online version contains supplementary material available at 10.1186/s43897-023-00071-2.

## Core

The expression of transcription factors CsMYB123 and CsbHLH111 were increased under drought stress, and CsMYB123 interacts with CsbHLH111 to positively regulate the expression of *CsCHI*, which controls anthocyanins biosynthesis, through binding to its promoter, thus regulating the biosynthesis of anthocyanins.

### Gene and accession numbers

Sequence data from this article can be found in the database of the National Center for Biotechnology Information (NCBI) under the accession numbers: CsMYB123: OR198176, CsbHLH111: OR198177, PbbHLH111: XP048429068.1, PdbHLH111: XP007222467.1, PabHLH111(*Prunus avium*): XP021821068.1, PabHLH111 (*Potentilla anserina*): XP050373164.1, BnbHLH111: XP013659422.1, CabHLH111: XP016537422.2, PgbHLH111: XP031395449.1, EgbHLH111: XP010043891.1, RabHLH111: XP030512787.1, MpbHLH111: RDX65597.1, VvbHLH111: XP002268359.2, QrbHLH111: XP050275725.1, ZjbHLH111: XP048318181.1, CsbHLH111 (*Camellia sinensis*): XP028080241.1

## Introduction

*Chaenomeles speciosa*, a shrub of *Chaenomeles* in the Rosaceae family, is a highly regarded ornamental plant extensively utilized in landscapes and gardens. However, the early flowering period of *C. speciosa* resulted in a significant decrease in its ornamental value after the flowering phase. Thus, enhancing the aestetic appeal of foliage holds substantial importance in prolonging the ornamental duration of *C. speciosa*.

The primary determinant of the ornamental value of leaves is color, and anthocyanin is the principal contributor to the vibrant hues of numerous plant leaves and flowers (Landi et al. 2015). The biosynthesis of plant-derived anthocyanin was subject to multifarious factors, such as drought stress (Zhang et al. 2022). In response to drought stress, plants exhibited augmented anthocyanin biosynthesis, thereby efficiently mitigating the build-up of stress-induced reactive oxygen species (ROS) and preserving cellular integrity vital for plant survival (Kaur et al. 2023). Currently, the phenomenon of drought stress-induced anthocyanin synthesis in plants is well-known; however, it was reported that its molecular mechanism was few, only such as *Solanum tuberosum*, *Malus pumila*, and *Solanum lycopersicum*, and remains elusive (André et al. 2009; An et al. 2020; Tapia et al. 2022). Therefore, revealing the mechanism of anthocyanidin biosynthesis under drought stress was of great significance for understanding the secondary metabolism process of plants under stress and improving the ornamental value of plants.

The biosynthesis of anthocyanins involves the synergistic interaction between transcription factors and cis-regulatory elements in the promoter regions of structural genes, such as *CHI*, *CHS*, *ANS*, *UFGT*, *DFR*, and *F3H* (Tanaka et al. 2008, Li et al. 2016, Chaves-Silva et al. 2018, Ma, Constabel 2019). The transcription factors that regulate anthocyanin biosynthesis primarily include MYB, bHLH and WD40 (Gonzalez et al. 2008). Furthermore, increasing evidence indicates that the expression of the members of the MBW complex, specifically MYBs and bHLHs, is modulated by environmental temperatures and other stimuli, suggesting a novel role for the MBW regulatory complex in response to environmental effects on anthocyanin synthesis in plants (Ban et al. 2007, Rowan et al. 2009, Lin-Wang et al. 2011). However, the molecular mechanism by which MBW components regulate anthocyanin biosynthesis and organ coloration in response to environmental stimuli is largely unknown. MYB, especially the R2R3-MYB transcription factor, Specific binding to target gene DNA is a major determinant of the spatial and temporal accumulation characteristics of anthocyanin glycosides (Albert et al. 2010; Yan et al. 2021), such as MdMYB10 in apple, DcMYB7 in *Daucus carota*, and LrMYB15 in *Lilium regale* (Espley et al. 2007; Xu et al. 2019; Yin et al. 2021). Therefore, identifying the R2R3-MYB gene, which regulates anthocyanin biosynthesis in ornamental plants, is particularly important for unraveling the transcriptional regulatory mechanism of color formation in them. bHLH, as the second-largest transcription factor family in plants, was also involved in regulating anthocyanin synthesis and stress responses (Feller et al. 2011). For example, the bHLH transcription factor BrTT8 promotes anthocyanin synthesis in turnip under abiotic stress (Zhang et al. 2020a), and MdbHLH3 in apple enhanced anthocyanin accumulation in fruits under low-temperature stress (Xie et al. 2012). It has been shown that the regulation of phycocyanin by bHLH is mainly accomplished by means of interactions with other transcription factors. Furthermore, more studies have shown that bHLH can influence anthocyanin accumulation by interacting with MYB transcription factors (Zhao et al. 2019). In *Dendrobium* sp., the transcription factors DhMYB2 and DhbHLH1 are co-expressed with anthocyanin biosynthesis genes DhDFR and DhANS in petals to regulate anthocyanin synthesis (Li et al. 2017). In red pear (*Pyrus*), PyWRKY26 could interact with PybHLH3 and could bind to the PyMYB114 promoter and activate the transcription of PyMYB114, which results in anthocyanin accumulation in red-skinned pear (Chuang L et al. 2020). Currently, the regulatory role of bHLH in anthocyanin has yielded results in fruit trees, but studies in ornamental flowers have been less involved.

In this study, we revealed that CsMYB123 and CsbHLH111 are important promoters of anthocyanin biosynthesis under drought stress, using leaves of *C. speciosa* as materials. We confirmed that CsMYB123 and CsbHLH111 interacted and activated the promoter activity of the structural gene *CsCHI* to promote anthocyanidin accumulation by a variety of experimental methods, including transient expression assay, Y1H, dual-luciferase reporter assay, Y2H, and BIFC assays. Our findings aim to provide a foundation for the molecular investigation of anthocyanin synthesis regulation under drought stress.

## Results

### Drought stress promotes the accumulation of anthocyanins in the leaves of C. speciosa

**Fig. 1:**
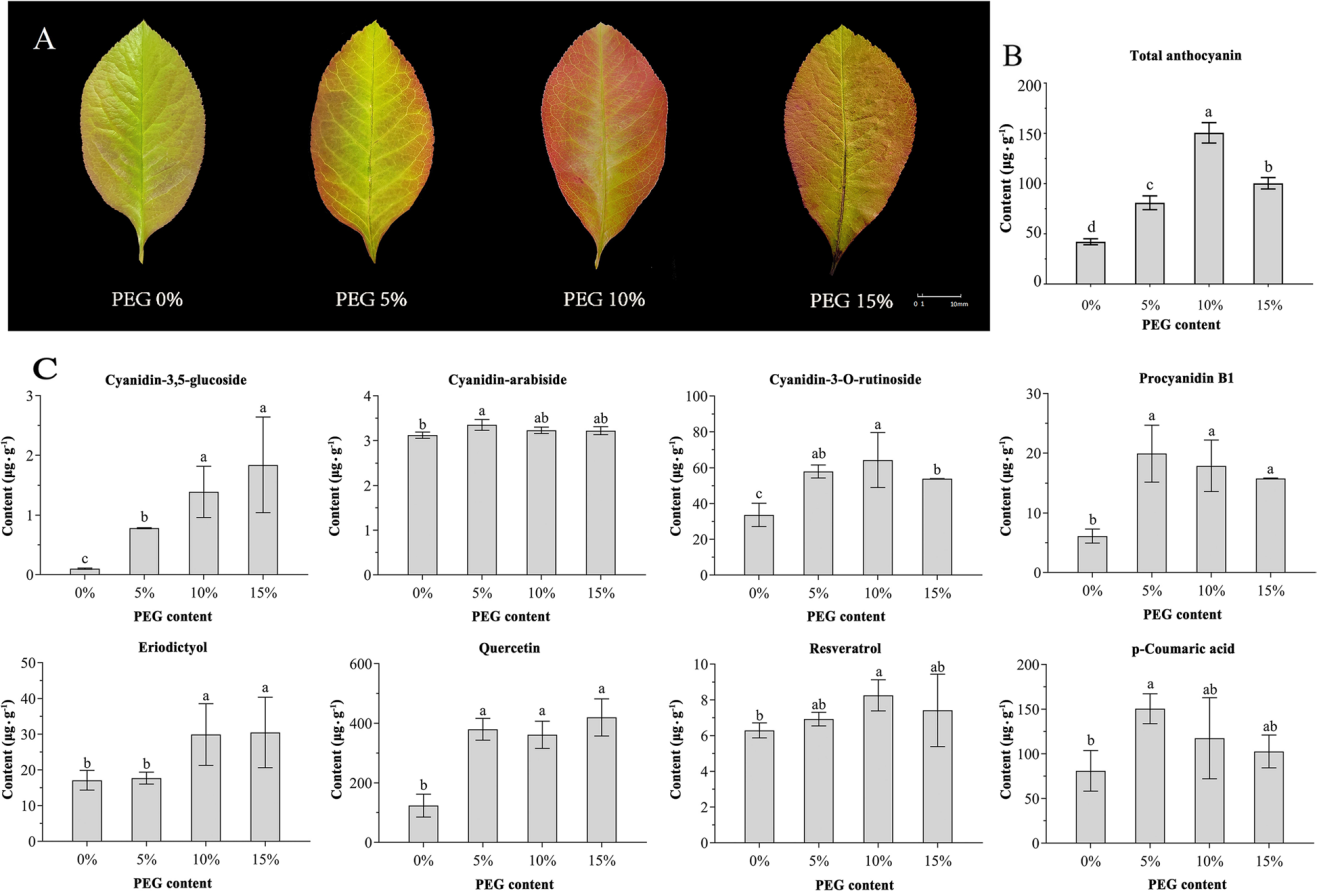
Changes of leaf color and Substance contents of *C. speciosa* under different concentrations of drought stress. **A** Changes in the phenotype of leaves treated with 0–15% PEG-8000; **B**: Changes in total anthocyanin content under different concentrations of PEG treatment. **C** Types and contents of anthocyanin biosynthesis-related compounds

Distinct color changes were observed in the leaves of *C. speciosa* under different concentrations of PEG-8000. As the PEG content increased, the leaf color gradually deepened (Fig. 1A). Total anthocyanin was extracted and quantified using UV spectrophotometry (Fig. 1B). The results revealed that the anthocyanin content increased with the rise in PEG concentration. Specifically, the highest anthocyanin content of 150.37 ± 8.49 μg·g^−1^ was recorded at a PEG concentration of 10%.

The present study employed high-performance liquid chromatography to separate and quantitatively determine the content of anthocyanins and related compounds in the leaves of *C. speciosa*. By comparing the determination results of HPLC–DAD with standard UV absorption curves and retention times, the specific types of anthocyanins and related compounds present in *C. speciosa* were identified (Fig. 1C). Three anthocyanins, namely Cyanidin-3,5-glucoside, Cyanidin-arabiside, and Cyanidin-3-*O*-rutinoside, were detected at 520 nm. Proanthocyanidin B1 was detected at 280 nm. Additionally, the presence of resveratrol, p-coumaric acid, quercetin, and eriodictyol were observed in the samples. Quantification of each substance was performed using the respective standard curves. The results indicated that the main compound responsible for the deepening of leaf color in *C. speciosa* under drought stress was Cyanidin-3-*O*-rutinoside, and its variations in content were consistent with the changes in leaf color observed under drought stress-induced. Additionally, the contents of proanthocyanidin B1, quercetin, and p-coumaric acid in the leaves of *C. speciosa* appeared to increase with the deepening of drought. Among these, proanthocyanidin B1 and quercetin are both flavonoids, which are important for plant resistance to damage.

### Screening and cloning of anthocyanin-related genes

**Fig. 2:**
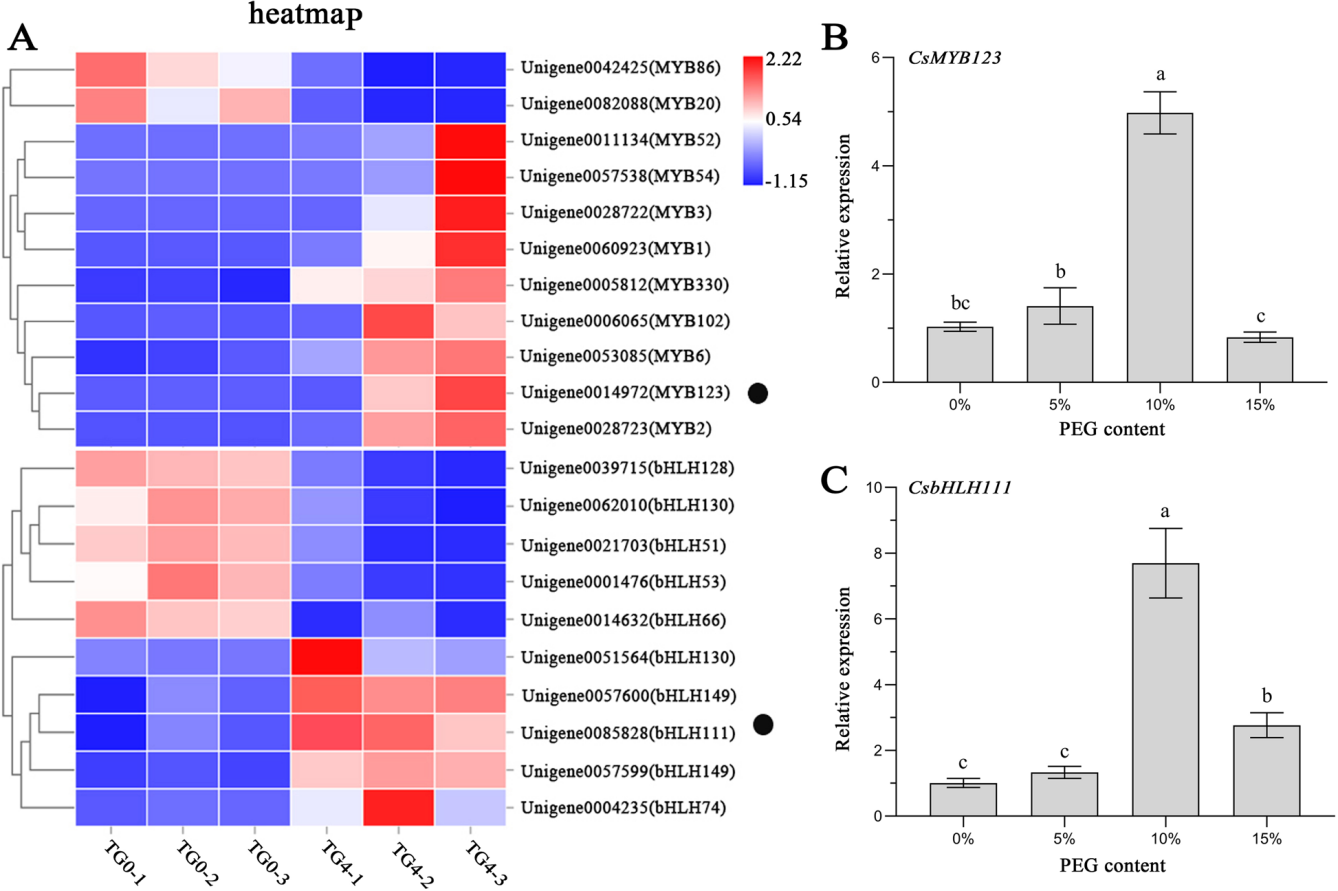
Screening genes. **A** Heat map of Genes (TG0: 0% PEG-8000, TG4: 4% PEG-8000; 1, 2, and 3 refer to the three repetitions). **B** The expression analyses of *CsMYB123* in *C. speciosa* with different PEG concentrations. **C** The expression analyses of *CsbHLH111* in *C. speciosa* with different PEG concentrations

Through the transcriptomic data obtained under drought stress in flowers of *C. speciosa*, 20 differentially expressed DEGs belonging to MYB and bHLH were screened (Fig. 2A). The qRT-PCR analysis of the up-regulated DEGs among differentially expressed was performed using cDNAs of *C. speciosa* leaves treated with different PEG contents. The analysis revealed a distinct pattern which was the same as the changing trend of anthocyanin content in the relative expression levels of *CsMYB123* and *CsbHLH111* with increasing PEG concentration (Fig. 2B, C). However, gene expression of MYB330, MYB102, MYB6, MYB2, and bHLH149 did not show the same results (Fig. S1).

Specific primers were designed based on the transcriptomic sequences for cloning of *CsMYB123* and *CsbHLH111* using cDNA from the leaf tissues of *C. speciosa*. The obtained *CsMYB123* coding sequence (CDS) was found to be 984 bp in length, encoding 327 amino acids, while the *CsbHLH111* CDS spanned 1629 bp, encoding 547 amino acids.

**Fig. 3:**
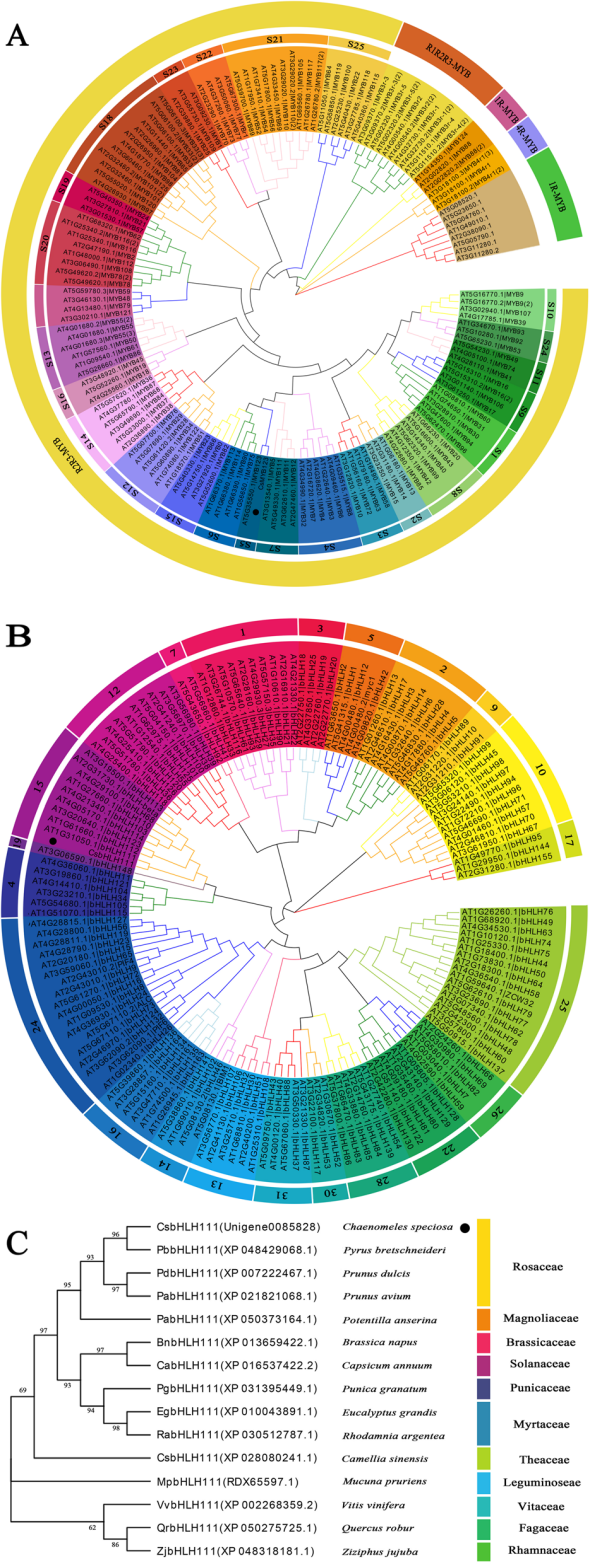
Phylogenetic tree analysis. **A** Phylogenetic tree analysis of CsMYB123 and 146 *Arabidopsis thaliana* proteins. The phylogenetic tree was constructed using the Neighbor-joining method with 1000 bootstrap replications*.* **B** Phylogenetic tree analysis of CsbHLH111 and 147 *Arabidopsis thaliana* proteins. **C**: Phylogenetic tree analysis of bHLH111 protein from *C. speciosa* and 14 other species

Using the approach of neighbor-joining, we constructed phylogenetic trees of 146 MYB proteins and 147 bHLH proteins from *Arabidopsis thaliana*, based on cloned *CsMYB123* and *CsbHLH111* sequences. Subgroups were designated according to the clustering results (S1-S25) of the earlier study in *Arabidopsis* (Dubos et al. 2010). The CsMYB123 and AtMYB123 proteins cluster together, both belonging to the S5 subgroup of R2R3 transcription factor associated with anthocyanin biosynthesis (Fig. 3A). Similarly, CsbHLH111 and AtbHLH111 proteins form a distinct cluster within the 15th subgroup according to the clustering results of the earlier study (Fig. 3B) (Carretero-Paulet et al. 2010).

A phylogenetic tree was constructed using MEGA11.0 software based on the protein sequences of bHLH111 from 14 different species. The analysis revealed that the CsbHLH111 protein sequence clustered together with the bHLH111 protein sequence from *Pyrus bretschneideri*. Additionally, bHLH111 of *Prunus dulcis* and bHLH111 of *Prunus avium* were found to be closely related to each other (Fig. 3C).

### Subcellular localization of CaMYB123 and CsbHLH111

To investigate where CsMYB123 and CsbLHH111 proteins exert functions, constructs with CsMYB123 or CsbHLH111 infused with green fluorescent protein (GFP) were introduced into Arabidopsis protoplasts to determine subcellular localization. The experimental results showed that CsMYB123 was localized to the nucleus, whereas CsbHLH111 was localized to the cytoplasm and nucleus (Fig. S2).

### Transient overexpression of CsMYB123 and CsbHLH111 promotes anthocyanin biosynthesis

**Fig. 4:**
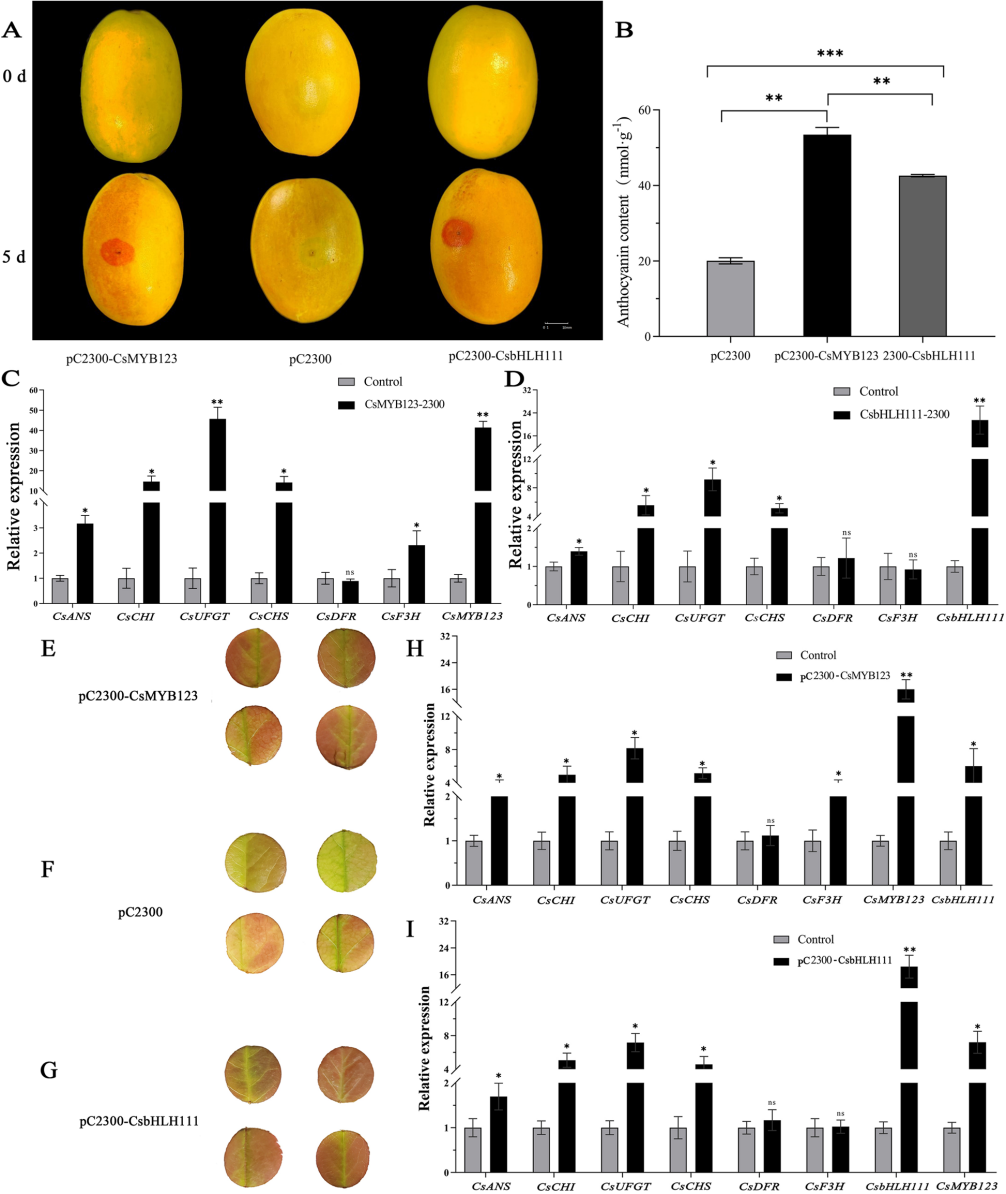
*CsMYB123* and *CsbHLH111* were overexpressed in peels and leaves of *Chaenomeles*. **A** Phenotypic changes in peels after over-expression of *CsMYB123* and *CsbHLH111*. **B** Changes in total anthocyanin content. **C** Changes in the relative expression of structural genes in peels of overexpressing *CsMYB123*. **D** Changes in the relative expression of structural genes in peels of overexpressing *CsbHLH111*. **E** Phenotypic changes in leaves after over-expression of *CsMYB123.* **F** The control of over-expression of *CsMYB123* and *CsbHLH111* in leaves of *Chaenomeles.* **G** Phenotypic changes in leaves after over-expression of *CsbHLH111.* **H** Changes in the relative expression of structural genes in leaves of overexpressing *CsMYB123.* **I** Changes in the relative expression of structural genes in leaves of overexpressing *CsbHLH111*

To investigate the functions of the transcription factors CsMYB123 and CsbHLH111, constructed pC2300-CsMYB123 and pC2300-CsbHLH111 Agrobacterium suspensions were introduced into peels and leaves of *Chaenomeles* for transient overexpression analysis, with pC2300 Agrobacterium suspension serving as the control. The results demonstrated that the peel and leaves injected with the recombinant overexpression vectors exhibited a deeper red coloration compared to the control (Fig. 4A, E, F, G). Furthermore, the quantification of anthocyanin content revealed that the transient overexpression of pC2300-CsMYB123 and pC2300-CsbHLH111 resulted in a significant increase in anthocyanin content compared to the control (Fig. 4B).

The qRT-PCR analysis was performed on the transiently overexpressed peels and leaves (Fig. 4C, D, H, I). The results revealed a significant enhancement in the relative expression levels of the *CsMYB123* in the peels and leaves of overexpressing *CsMYB123*, and the expression levels of the key structural genes *CsCHI*, *CsANS*, *CsUFGT*, *CsCHS*, and *CsF3H* were significantly upregulated. In peels overexpressing *CsbHLH111*, a significant enhancement in the expression level of the *CsbHLH111* was observed, exhibiting a 21.5-fold increase compared to the control. Additionally, the relative expression levels of *CsCHI*, *CsUFGT*, and *CsCHS* were upregulated. Interestingly, when we transiently overexpressed *CsMYB123* in leaves of *Chaenomeles speciosa*, gene expression of *CsbHLH111* was similarly increased (Fig. 4H).

### The VIGS of CsMYB123 and CsbHLH111 reduce anthocyanin accumulation

**Fig. 5:**
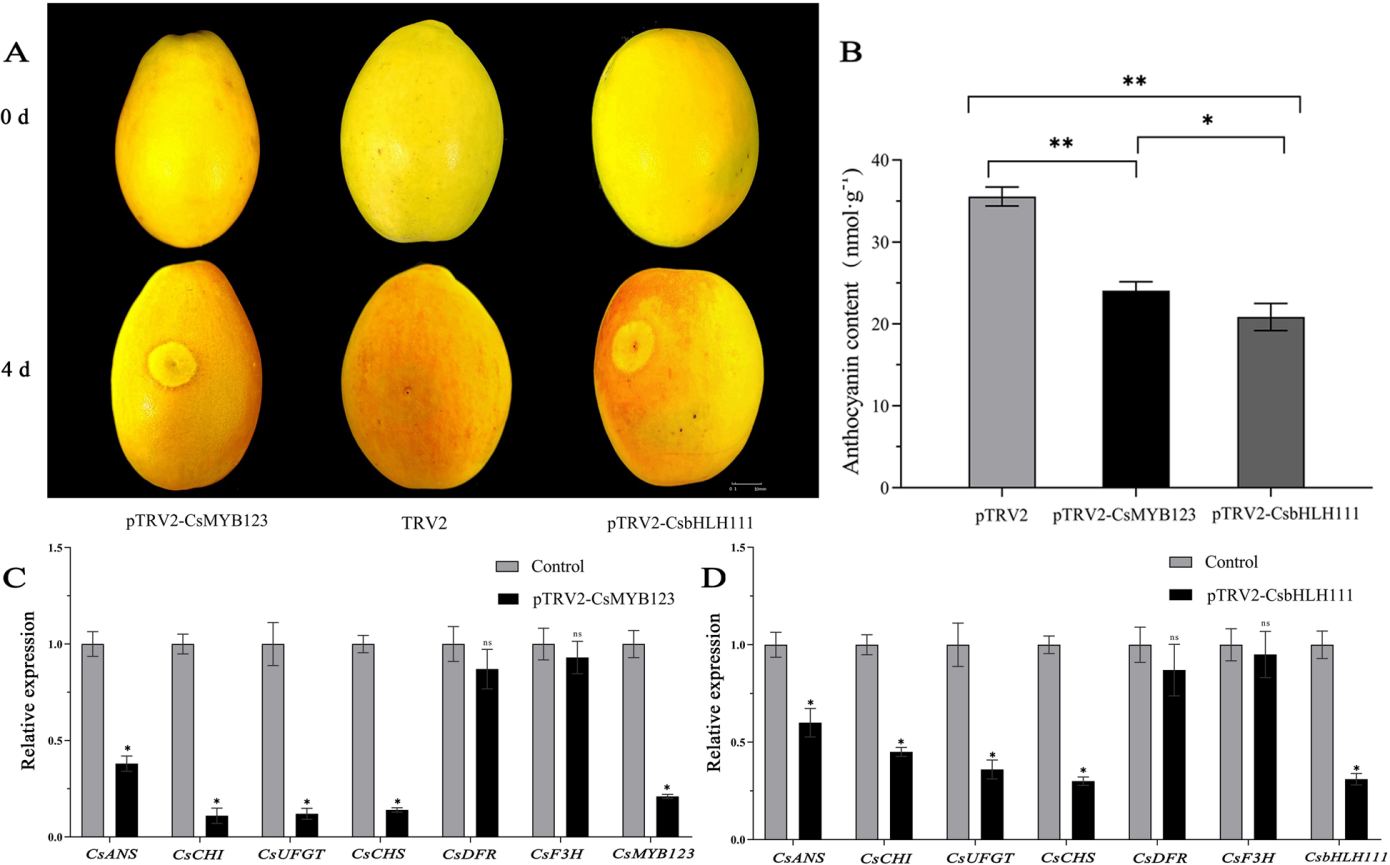
*CsMYB123* and *CsbHLH111* were overexpressed in peels. **A** Phenotypic changes in peels after VIGS of *CsMYB123* and *CsbHLH111*. **B** Changes in total anthocyanin content. **C** Changes in the relative expression of the key structural genes after VIGS of *CsMYB123* in *Chaenomeles*. **D** Changes in the relative expression of the key structural genes after VIGS of *CsbHLH111* in *Chaenomeles*

To further investigate the role of transcription factors CsMYB123 and CsbHLH111 in anthocyanin synthesis, the constructed TRV2-CsMYB123 and TRV2-CsbHLH111 vectors were transiently silenced in *Chaenomeles*, with TRV2 empty vector as the control. After 4 days, the VIGS of *CsMYB123* or *CsbHLH111* exhibited reduced red color intensity in the fruits compared to the control (Fig. 5A). The total anthocyanin content in each sample revealed a significant decrease when *CsMYB123* or *CsbHLH111* was silenced (Fig. 5B).

The results of qRT-PCR analysis showed a significant decrease in the expression levels of *CsMYB123* and the key structural genes *CsCHI*, *CsANS*, *CsUFGT*, and *CsCHS*, when compared to the control. Similarly, a significant reduction was observed in the expression levels of *CsCHI*, *CsANS*, *CsUFGT*, and *CsCHS* upon the VIGS of *CsbHLH111* (Fig. 5C, D).

### CsMYB123 and CsbHLH111 bind to promoters of structural genes related to anthocyanin biosynthesis

**Fig. 6:**
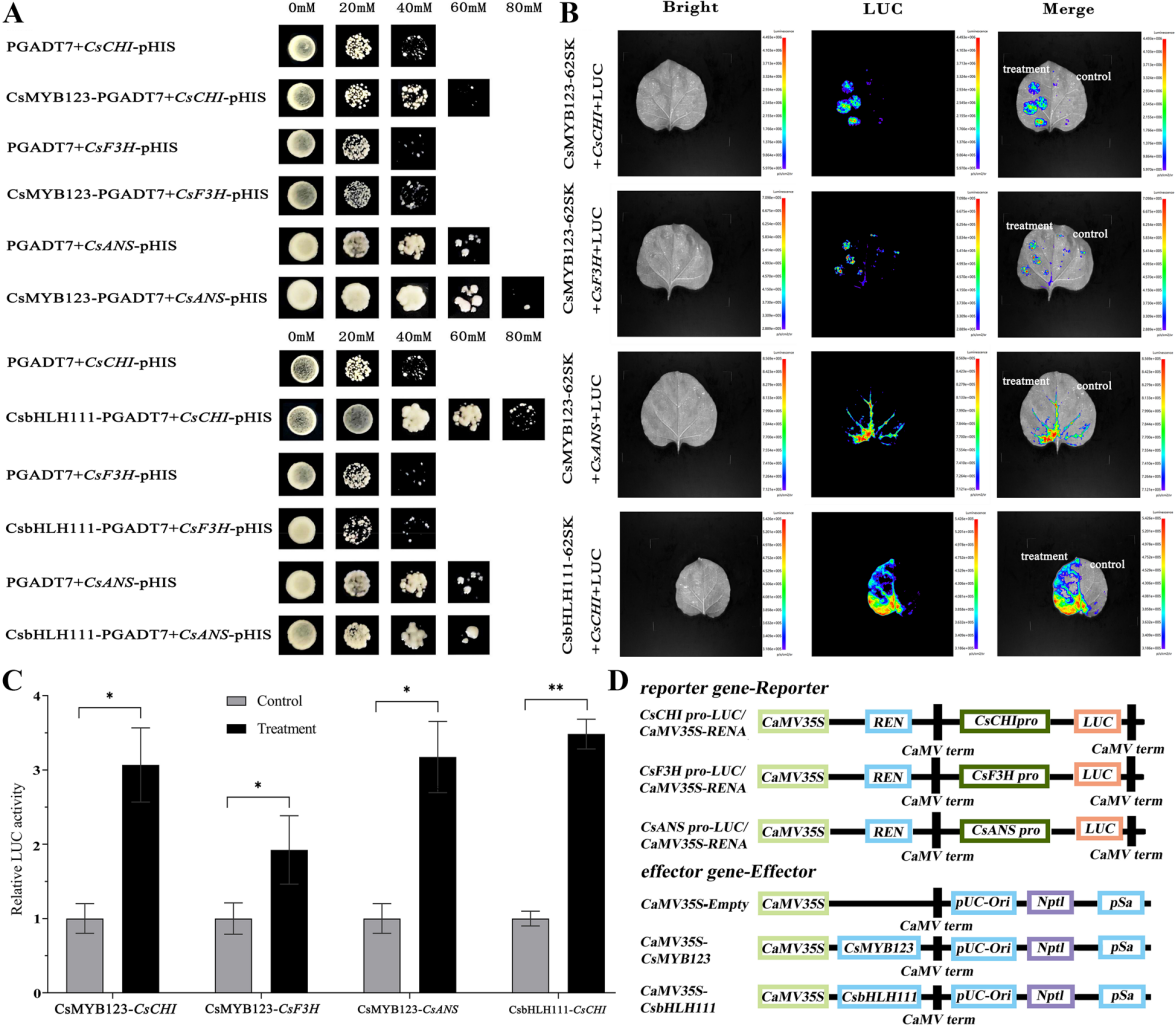
*CsMYB123* and *CsbHLH111* promote the transcriptional activity of *CsCHI*, *CsF3H* and *CsANS*. **A** The result of Y1H. **B** Dual-luciferase reporter assay (the treatment on the left side of the leaves, control on the right side). **C** The activity determination of *CsMYB123* and *CsbHLH111* LUC/REN. **D** Schematic representations of the vectors used for luciferase assay

Transcription factors play a crucial role in regulating anthocyanin synthesis by binding to the promoters of structural genes. To investigate the regulatory mechanisms of *CsMYB123* and *CsbHLH111* in anthocyanin biosynthesis, Y1H assays were used to examine the interactions between the transcription factors and structural genes. The experimental results revealed that the Y187 yeast strain co-transformed with CsMYB123-pGADT7 and *CsCHI*-pHIS2, *CsANS*-pHIS2, or *CsF3H*-pHIS2 exhibited enhanced activity compared to the control on SD/-T/-H/-A medium. Moreover, the Y187 yeast strain co-transformed with CsbHLH111-pGADT7 and *CsCHI*-pHIS2 showed increased activity compared to the control (Fig. 6A).

The analysis conducted using the Dual-luciferase reporter assay unveiled significant findings of importance (Fig. 6B). The expression of *CsMYB123* resulted in a significant increase in the LUC activity of *CsANS*, *CsCHI*, and *CsF3H* promoters. Similarly, upon the expression of CsbHLH111, there was a notable enhancement in the LUC activity of the *CsCHI* promoter (Fig. 6C, D).

### CsMYB123 interacts with the CsbHLH111 protein on the cell nucleus

**Fig. 7:**
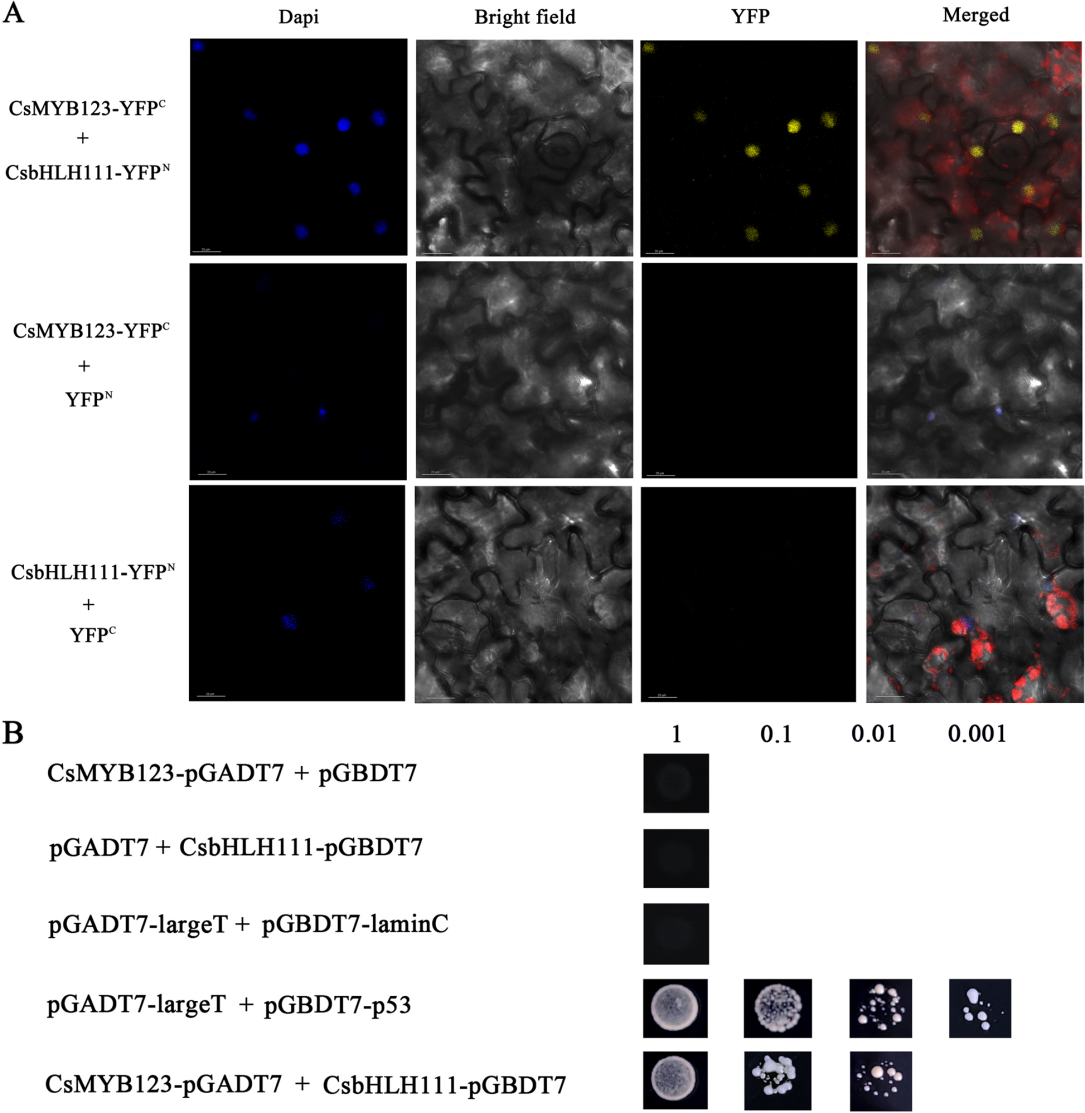
CsMYB123 interacts with CsbHLH111. **A** Bimolecular fluorescence complementation diagram of CsMYB123 and CsbHLH111. **B** Yeast two-hybrid assays (Y2H assays) showing that CsMYB123 can interact with CsbHLH111 in yeast

The results from the BiFC assay demonstrated an intriguing pattern (Fig. 7A). In tobacco leaf cells co-transformed by CsMYB123-YEP^C^ and CsbHLH111-YEP^N^ plasmids, a distinct yellow fluorescence signal was observed. The results by Dapi staining showed that its yellow fluorescence was located on the nucleus of the cells. In addition, the result of Y2H showed that yeast in which CsMYB123 was cotransformed with CsbHLH111 showed higher activity compared to the control (Fig. 7B). These findings indicate a potential interaction between CsMYB123 and CsbHLH111 proteins occurring at the cell *nucleus*.

## Discussion

Drought stress exerts significant effects on plant anthocyanin synthesis. In this study, an accumulation of anthocyanin was observed upon PEG treatment mimicking drought stress in the leaves of *C. speciosa*. The results of HPLC–DAD indicated that the main compound responsible for the change of color in leaves was Cyanidin-3-*O*-rutinoside under drought stress. However, interestingly, different plant species employed distinct key transcription factors to regulate anthocyanin biosynthesis in response to drought stress. For instance, the ERF transcription factor MdERF38 was induced by drought stress to promote anthocyanin synthesis in apples, while SpAN2 played a crucial role in anthocyanin biosynthesis under drought-include in *Solanum pimpinellifolium* (An et al. 2020; Tapia et al. 2022). In this study, *CsMYB123* and *CsbHLH111* were screened and isolated through transcriptome and qRT-PCR analysis of gene expression in leaves treated with different PEG concentrations. The phylogenetic tree analysis a close relationship between CsMYB123 and AtMYB123 from the S5 subfamily, which is associated with anthocyanin biosynthesis in Arabidopsis. This suggested that *CsMYB123* might be involved in the synthesis of anthocyanins in *C. speciosa*. Previous research has shown that plum *bHLH111* upregulates the expression of structural genes involved in anthocyanin biosynthesis at intermediate storage temperatures, contributing to the accumulation of anthocyanins in the fruit flesh of the “Friar” plum variety (Li et al. 2021). Interestingly, our research discovered that *CsbHLH111* shares a close phylogenetic relationship with plum *bHLH111*. Subsequent transient overexpression experiments demonstrated that *CsMYB123* and *CsbHLH111* positively controlled anthocyanin biosynthesis in *C. speciosa*. Moreover, the VIGS of gene experiments revealed the crucial roles of *CsMYB123* and *CsbHLH111* in anthocyanin synthesis.

It is noteworthy that the regulatory mechanisms of the same transcription factors involved in anthocyanin biosynthesis vary among different species. For instance, AcMYB123 plays a crucial component in anthocyanin synthesis by activating the *AcANS* promoter in kiwifruit peel (Wang et al. 2019). Similarly, in *Liquidambar formosana*, LfMYB123 promotes anthocyanin accumulation by regulating *LfF3´H* and *LfF3´5´H* (Wen et al. 2021). While in *Prunus persica*, PpMYB123 enhances anthocyanin synthesis by upregulating *PpUFGT* (Ravaglia et al. 2013). Here, Y1H assay and Dual-luciferase reporter assay revealed that CsMYB123 interacts with the promoters of *CsCHI*, *CsF3H*, and *CsANS*, positively regulating their activity and thereby promoting anthocyanin biosynthesis.

Several studies demonstrated that bHLH transcription factors can directly regulate structural genes. PsbHLH1 directly interacts with the promoters of anthocyanin biosynthesis genes *PsDFR* and *PsANS*, upregulating their expression during flower development and pigment accumulation of *Paeonia suffruticosa* (Qi et al. 2020). The CmbHLH2 binds to the *CmDFR* promoter, regulating anthocyanin biosynthesis in *Chrysanthemum morifolium* Ramat (Xiang et al. 2015). In this study, the Y1H assay revealed that CsbHLH111 interacts with the *CsCHI* promoter. Furthermore, dual-luciferase assays demonstrated a significant increase in LUC activity driven by the *CsCHI* promoter when CsbHLH111 was expressed. These findings indicated that the transcription factor CsbHLH111 directly binds to the *CsCHI* promoter and positively regulates anthocyanin biosynthesis in *C. speciosa*.

In plants, the bHLH usually interacts with MYB proteins, jointly regulating anthocyanin biosynthesis. For instance, in *Actinidia deliciosa*, AcbHLH42 interacts with AcMYB10, promoting anthocyanin biosynthesis in the fruit (Yu et al. 2019). In *Populus simonii*, the bHLH protein PdTT8 directly interacts with PdMYB118, promoting anthocyanin synthesis in wounded poplar tissues (Wang et al. 2020). In *Phalaenopsis aphrodite*, the bHLH protein PeMYC4 interacts with PeMYB4L to downregulate the expression of *PeCHI*, inhibiting anthocyanin accumulation (Wang et al. 2022). In our study, the results of subcellular localization showed that CsMYB123 was localized in the nucleus, while CsbHLH111 was localized in both the cytoplasm and the nucleus, which opens up the possibility of interactions between CsMYB123 and CsbHLH111. In addition, the results of Y2H and the BIFC assay preliminarily suggested that an interaction between CsMYB123 and CsbHLH111 proteins occurs at the cell nucleus. However, the regulatory mechanisms following anthocyanin protein interactions under drought stress need to be further investigated.

In summary, our research indicated that drought stress contributes to the promotion of anthocyanin accumulation, particularly Cyanidin-3-*O*-rutinoside, in the leaves of *C. speciosa*. Furthermore, CsMYB123 interacts with CsbHLH111 to improve the activity of the structural gene *CsCHI* promoter, which regulates the anthocyanins biosynthesis in *C. speciosa* under drought stress, leading to reddening of the leaves. These findings contribute to our understanding of the intricate regulatory mechanisms underlying anthocyanin synthesis in response to drought stress and shed light on the species-specific variations in transcriptional regulation of this process in plants.

### Materials and methods

#### Plant materials and experimental design

Plant materials were obtained from the campus of Northwest A&F University in Yangling District, Xianyang City, Shaanxi Province, China. The attached petioles of *C. speciosa* leaves were subjected to drought stress simulation using PEG-8000 at concentrations of 0%, 5%, 10%, and 15% for a duration of 48 h, with three biological replicates per concentration gradient. Subsequently, the treated leaves were rapidly frozen in liquid nitrogen and stored at -80 °C for subsequent analyses, including determination of anthocyanin content, gene cloning, and fluorescence quantification.

### Measurement of total anthocyanin contents

Approximately 0.25 g of treated samples were placed in a mortar and ground in liquid nitrogen. The resulting powder was transferred to a 10 mL centrifuge tube, to which 5 mL of methanol solution was added. The tube was then placed in darkness at 4 °C and gently inverted 2–3 times every 12 h. After 48 h, the mixture was centrifuged at 10,000 rpm for 10 min.

Total anthocyanin contents were determined by methanol hydrochloric acid method (Li et al., 2012). Take 800 μL of extraction solution and add 24 μL of HCl. Add 24 μL of HCl to the extraction solution and mix well. After mixing, the reaction was carried out at room temperature and protected from light for 15 min, and the absorbance of the reaction solution was measured at 530 nm, 620 nm, and 650 nm using an enzyme meter. The anthocyanin content was quantified using the following formula: OD = (OD_530_-OD_620_)-0.1 × (OD_650_-OD_620_). One unit of anthocyanin content was expressed as a change of 0.1 OD (unit × 10^3^·g^−1^ FW).

### High-Pressure Liquid Chromatography Analysis

After grinding 0.5 g of leaves into powder, it was added to a 10 mL solution of pure methanol, and the extraction process was conducted at 4 °C in a light-free environment for 48 h. The resulting mixture was subjected to ultrasonic extraction for 20 min and then centrifuged at 8000 rpm for 10 min to collect the supernatant. The supernatant was concentrated using a rotary evaporator at 38 °C until dryness. Subsequently, 1 mL of pure methanol solution was added to wash the walls of the vial, and the solution was extracted using a sterile syringe. The extract was then injected into sample vials and stored for future use, following filtration through a 0.22 μm organic membrane filter and analyzed using High-Performance Liquid Chromatograph L-2455 Diode Array Detector (Shimadzu, Japan).

The mobile phase A consisted of a 0.04% formic acid aqueous solution, while the mobile phase B consisted of pure acetonitrile. The temperature was set at 30 °C, and the flow rate was maintained at 1.0 mL·min^−1^. The solvent gradient employed in this study was as follows: 0 min—95%A, 5%B; 25 min—85%A, 15%B; 42 min—78%A, 22%B; 60 min—64%A, 35%B; 65 min—95%A, 5%B. The column temperature was maintained at 40 °C, and the flow rate was adjusted to 0.5 mL·min^−1^. The injection volume was set at 10 μL, and the detection wavelengths were 280 nm, 360 nm, and 520 nm. Compound identification was performed by comparing the retention times and UV spectra data with those of authentic standards (Liu et al. 2019, Han et al. 2020).

### Total RNA extraction and cDNA synthesis

Total RNA extraction from the treated samples was performed using the TianGen RNA extraction kit. The RNA content was measured, and subsequently, reverse transcription was carried out to synthesize cDNA. Based on the transcriptome data of *C. speciosa* under drought stress (available at https://www.ncbi.nlm.nih.gov/sra/PRJNA905705), genes exhibiting differential expression were selected and primers were designed accordingly. qRT-PCR technique was employed to analyze the genes associated with anthocyanin accumulation in response to drought conditions.

### Evolutionary tree analysis

The protein sequences of MYB and bHLH from *Arabidopsis thaliana* were obtained from PlantTFDB. Additionally, the bHLH111 protein sequences from 14 different species were downloaded from the NCBI database. Evolutionary analysis of these sequences was performed using MEGA11.0 software, and a phylogenetic tree representing the systematic evolutionary relationships of CsMYB123, and CsbHLH111 with Arabidopsis thaliana was constructed using the neighbor-joining method. In addition, a phylogenetic tree of bHLH111 protein was constructed with 14 different species using the Maximum likelihood method. To enhance the visual presentation of the evolutionary tree, the Evolview online platform (https://evolgenius.info//evolview-v2/#login) was utilized.

### Subcellular localization assay

The cDNA sequences of *CsMY123* and *CsbHLH111* were cloned into the pSuper1300-GFP vector. The recombinant plasmid was transferred into GV3101. Tobacco leaves are injected after resuspension of Agrobacterium bacteria. After incubation under low-light conditions for 2 days, the injected portions were observed using laser confocal microscopy.

### Transient expression assays

The coding sequences (CDS) of *CsMYB123* and *CsbHLH111* were ligated into the pCAMBIA2300 vector and the 200–300 bp fragments at the 3´end of the *CsMYB123* and *CsbHLH111* coding sequences were ligated to the TRV2 vector. The pCAMBIA2300 or TRV1 was used as a helper vector. The resulting recombinant plasmids were then transferred into Agrobacterium tumefaciens GV3101. The resuspended bacterial culture was injected into the peels of *Chaenomeles* under dark conditions and incubated for 24 h. The peels were then transferred to a growth chamber with UV-B lamps under the following conditions: temperature of 25 °C, light intensity of 0%, and humidity of 75%. Samples were collected and stored at -80 °C for subsequent analyses, including determination of total anthocyanin content and qRT-PCR analysis. The primer sequences for plasmid construction are listed in Supplementary Table S1.

### Yeast one-hybrid (Y1H) assay

The coding sequences of *CsMYB123* and *CsbHLH111* were cloned into the pGADT7 vector, while the promoter sequences of structural genes *CsANS*, *CsF3H*, and *CsCHI* (already available in the laboratory) were cloned into the pHIS2 vector. The recombinant plasmids were cotransformed into yeasts and cultured on a screening medium lacking Trp and Leu (SD/–Trp–Leu) at 29 °C. Positive colonies were selected, and OD600 was adjusted to 0.1 using a 0.9% NaCl solution. Three biological replicates were performed. The yeast cells were then screened on a selective medium (SD/–Leu/–Trp/–His) containing the optimal concentration of 3-amino-1,2,4-triazole (3-AT). The primer sequences for plasmid construction are listed in Supplementary Table S1.

### Dual-luciferase reporter assay

The coding sequences of *CsMYB123* and *CsbHLH111* were cloned into the pGreen II 0029 62-SK vector, while the promoter sequences of *CsANS*, *CsF3H*, and *CsCHI* were cloned into the pGreen II 0800-LUC vector. The recombinant plasmids were separately transformed into tobacco GV3101 competent cells. The tobacco cultures containing the recombinant plasmids were resuspended and infiltrated into tobacco leaves. The infiltrated tobacco plants were incubated under low-light conditions for 2 days, and the activities of LUC and REN were measured. The promoter activities of structural genes *CsANS*, *CsF3H*, and *CsCHI* were calculated based on the ratio of LUC/REN. Three biological replicates were performed. The primer sequences for plasmid construction are listed in Supplementary Table S1.

### BIFC assays

The coding sequence of *CsMYB123* was ligated into the pSPYCE-ABF4 vector, while the CDS of *CsbHLH111* was ligated into the pSPYNE-CPK12 vector. The recombinant plasmids were separately transformed into tobacco GV3101 competent cells and cultured at 28 °C for 2 days. The tobacco cultures containing the recombinant plasmids were resuspended and adjusted to an optical density of 0.8 using a resuspension buffer. The cultures were mixed in a 1:1 ratio and infiltrated into tobacco leaves. After incubation under low-light conditions for 2 days, the injected portions were observed using laser confocal microscopy (Zhang H et al. 2020). The primer sequences for plasmid construction are listed in Supplementary Table S1.

### Yeast two-hybrid assay

The CDS sequences of CsMYB123 and CsbHLH111 without stop codon were inserted into pGADT7 or pGBDT7, respectively, to generate the CsMYB123-pGADT7 and CsbHLH111-pGBDT7 plasmids. The recombinant plasmids were transferred into the yeast strain Y2H Gold. The transformed yeast strains were grown on DO Supplement -Leu/-Trpmedium and DO Supplement -Ade/-His/-Leu/-Trp/ selective media at 28 °C for 3 days.

### Statistical analyses

The physiological parameters were expressed as three biological replicates' mean ± standard deviation. The obtained data were subjected to a one-way analysis of variance (ANOVA) using the Compare Means function in SPSS 26 software. The significance of differences was determined using the least significant difference (LSD) method for multiple comparisons. A *p*-value of less than 0.05 was considered statistically significant and significant differences of *P* < 0.05 (*) and *P* < 0.01 (**) were used for statistical studies. Data processing and graphical representation were performed using GraphPad software.

### Supplementary Information

**Additional file 1:  Supplementary Fig. S1. **The qPCR analysis of MYB330, MYB102, MYB6, bHLH149.

**Additional file 2: Supplementary Fig. S2. **Subcellular localization of CsMYB123 and CsbHLH111.

**Additional file 3: Supplementary Table S1. **Primer sequences.

## Data Availability

The authors confirm that all data in this study are included in this published article (and its supplementary information file).

## Abbreviations

MYB: Myeloblastosis
bHLH: Basic helix-loop-helix
BIFC: Bimolecular fluorescence complementation
PEG-8000: Polyethylene glycol-8000
HPLC–DAD: A diode array detector
qRT-PCR: Quantitative real-time polymerase chain reaction
DDO: Double dropout
LUC: Firefly luciferase
REN: Renilla luciferase
Y1H: Yeast one-hybrid
VIGS: Virus induced gene silencing
Y2H: Yeast two-hybrid
3AT: 3-Amino-1,2,4-triazole
