# Supplementary figures and images for: The CsMYB123 and CsbHLH111 are involved in drought stress-induced anthocyanin biosynthesis in Chaenomeles speciosa
Source: Mol Hortic. 2023 Nov 22;3:25. doi: 10.1186/s43897-023-00071-2 (PMC10664276; doi:10.1186/s43897-023-00071-2)

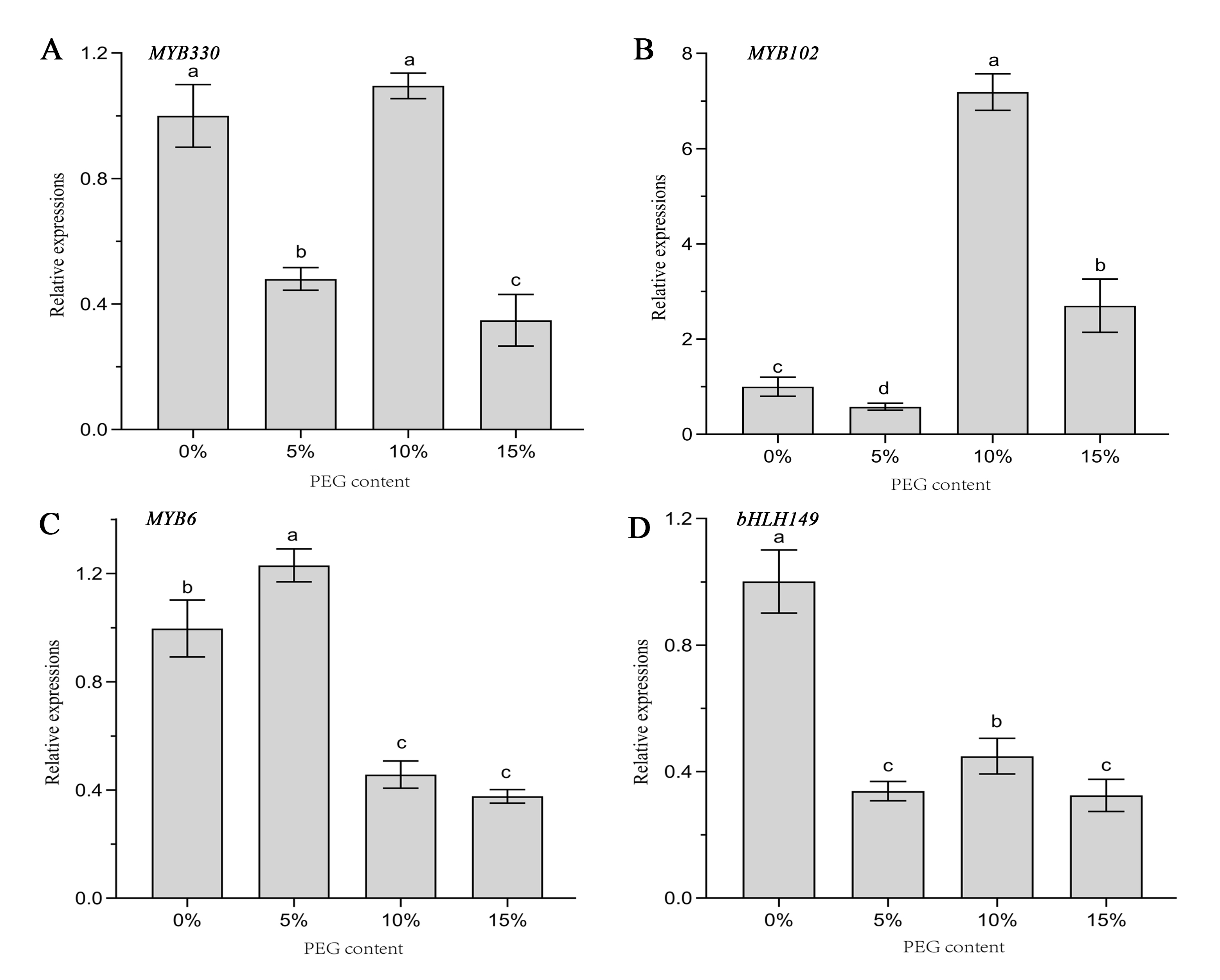

Supplement: Supplementary file 1 — Additional file 1: Supplementary Fig. S1. The qPCR analysis of MYB330, MYB102, MYB6, bHLH149. [file 43897_2023_71_MOESM1_ESM.png]

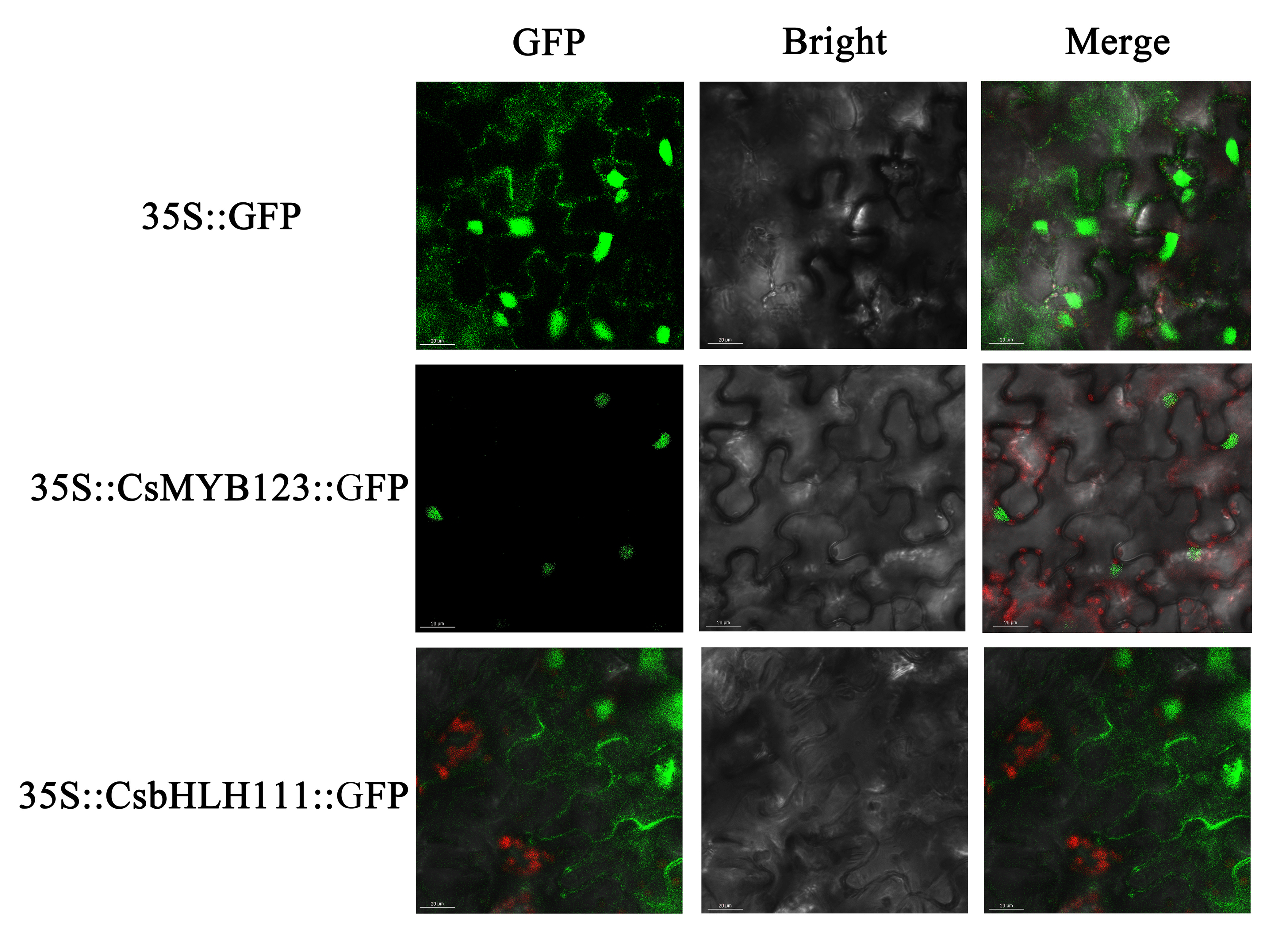

Supplement: Supplementary file 2 — Additional file 2: Supplementary Fig. S2. Subcellular localization of CsMYB123 and CsbHLH111. [file 43897_2023_71_MOESM2_ESM.png]
