# Supplementary material for: The CsMYB123 and CsbHLH111 are involved in drought stress-induced anthocyanin biosynthesis in Chaenomeles speciosa
Source: Mol Hortic. 2023 Nov 22;3:25. doi: 10.1186/s43897-023-00071-2 (PMC10664276; doi:10.1186/s43897-023-00071-2)
Supplement: Supplementary file 3 — Additional file 3: Supplementary Table S1. Primer sequences. [file 43897_2023_71_MOESM3_ESM.docx]

Supplementary table S1 primer sequences

| Primer name | Sequence（5–3′） |
| --- | --- |
| CsMYB123-F  CsMYB123-R  CsbHLH111-F  CsbHLH111-R  CsANS-F  CsANS-R  CsCHI-F  CsCHI-R  CsCHS-F  CsCHS-R  CsF3H-F  CsF3H-R  CsUFGT-F  CsUFGT-R  CsDFR-F  CsDFR-R  M13-47  M13-48  pC2300-F  pC2300-R  TRV2-F  TRV2-R  PGADT7-F  PGADT7-R  pHIS-F  pHIS-R  pGreenII 62-SK -F  pGreenII 62-SK -R  pGreenII 0800-LUC-F  pGreenII 0800-LUC-R  pSPYCE-F  pSPYCE-R  pSPYNE-F  pSPYNE-R  CsMYB123-F  CsMYB123-R  CsbHLH111-F  CsbHLH111-R  CsMYB123-（TRV2）-F  CsMYB123-（TRV2）-R  CsbHLH111-（TRV2）-F  CsbHLH111-（TRV2）-R  CsMYB123-（pC2300）-F  CsMYB123-（pC2300）-R  CsbHLH111-（pC2300）-F  CsbHLH111-（pC2300）-R  CsMYB123-（PGADT7）-F  CsMYB123-（PGADT7）-R  CsbHLH111-（PGADT7）-F  CsbHLH111-（PGADT7）-R  CsANS-（pHIS）-F  CsANS-（pHIS）-R  CsF3H-（pHIS）-F  CsF3H-（pHIS）-R  CsCHI-（pHIS）-F  CsCHI-（pHIS）-R  CsDFR-（pHIS）-F  CsDFR-（pHIS）-R  CsMYB123-（62-SK）-F  Cs MYB123-（62-SK）-R  CsbHLH111-（62-SK）-F  CsbHLH111-（62-SK）-R  CsANS-（800）-F  CsANS-（800）-R  CsF3H-（800）-F  CsF3H-（800）-R  CsCHI-（800）-F  CsCHI-（800）-R  CsMYB123-（pcambia1300）-F  CsMYB123-（pcambia1300）-R  CsbHLH111-（pcambia1300）-F  CsbHLH111-（pcambia1300）-RCsMYB123-（pSPYCE）-F  CsMYB123-（pSPYCE）-R CsbHLH111-（pSPYNE）-F  CsbHLH111-（pSPYNE）-R | ACATGGCATCACCATCATCCC  AGACTGGCTATGCTAATCTCGT  ATTGGTGGGATCTTGTCAGCT  ATCATGGTTTGCATTAGCGT  ATGTTGGAGAAGGACGACAC  AATCCGAATGGGACTGCAGG  GATTCCGTCATCACATTCCAT  TCTTGTCCCACCCAAATACCA  GGACGAAGTGAGGAGGAAGT  AAGCACAACGGTCTCAACAG  TGTAGCGGCTTGTGAGGACTG  TCCGATGGCAAAGCAAAGAAC  ATACATAGGTTCAAGCTCTCC  TGTACCATACTTGACCATCC  AGCAGGAACTGTGAATGTGGAG  GAGTTGGGATAATGGTGATGAAAT  CGCCAGGGTTTTCCCAGTCACGAC  AGCGGATAACAATTTCACACAGGA  AATGCAGCTGGCACGACAG  AACGACAATCTGATCCAAGCTC  AGTGGCTTGACGACACTAATG  ATGGTAAGACAATGAGTCGGC  AGTGGGAATATTGCTGATAGCTC ATGGTGCACGATGCACAGT  ATGTGCTGCAAGGCGATTAAGT  AGAGATCGCAATCTGAATCTTGGT  ATGTGACATCTCCACTGACGT  ACCTCTGACTTGAGCGTCGAT  ATTCGCCATTCAGGCTGCG  ATTCGATCTCCACCGCGGT  ATGACGCACAATCCCACTATCC  TGTGCTGCAAGGCGATTAAG  TGGTGAACCGCATCGAGCT  ATCATCGCAAGACCGGCAAC  ATGGGGAGAAGCCCTTGTTGT  ACGACTCTCTCCAACTCCAAG  ATGGCCGAAGAATGCAGTGTT  TCTATACAAACATCCTCTATATGTT  atggggatccggtacACGAGATTAGCATAGCCAGTCTTC  cttcgggacatgcccACGACTCTCTCCAACTCCAAG  aggttaccgaattctAGCCTGCTTGTGACGACTTGA  cttcgggacatgcccGTTGTGAGTACTGACGATCAT  attcgagctcggtacATGGGGAGAAGCCCTTGTTGT  ttgcatgcctgcaggACGACTCTCTCCAACTCCAAG  attcgagctcggtacATGGCCGAAGAATGCAGTGTT  ttgcatgcctgcaggTCTATACAAACATCCTCTATATGTT  agctcgagctcgatgATGGGGAGAAGCCCTTGTTGT  accagattacgctcaACGACTCTCTCCAACTCCAAG  agctcgagctcgatgATGGCCGAAGAATGCAGTGTT  accagattacgctcaTCTATACAAACATCCTCTATATGTT  actcactatagggcgaattcAGCCATTCCTCACAGGTACAAG  attcgcgaacgcgtgagctcAAACCCTTGTCTAAAATTT  actcactatagggcgaattcTCAATCGTCATGCCAAATAC  attcgcgaacgcgtgagctcATTGGTTCTGTCTACTTGGT  actcactatagggcgaattcTTTTAGGTTTTGGGGGCGCAC  attcgcgaacgcgtgagctcGTGTAGAGAGAAAGATGGGG  actcactatagggcgaattcAGGAAATCAACTACCACGCCAT  attcgcgaacgcgtgagctcTCTTGACCGAGCGGCAAAATT  aggaattcgatatcaATGGGGAGAAGCCCTTGTTGT  taccgaattggtacACGACTCTCTCCAACTCCAAG  aggaattcgatatcaATGGCCGAAGAATGCAGTGTT  taccgaattggtacTCTATACAAACATCCTCTATATGTT  gggcgaattgggtaccAGCCATTCCTCACAGGTACAAG  aggaattcgatatcaagcttAAACCCTTGTCTAAAATTT  gggcgaattgggtaccTCAATCGTCATGCCAAATAC  aggaattcgatatcaagcttATTGGTTCTGTCTACTTGGT  gggcgaattgggtaccTTTTAGGTTTTGGGGGCGCAC  aggaattcgatatcaagcttGTGTAGAGAGAAAGATGGGG  ccaaatcgactctagaATGGGGAGAAGCCCTTGTTGT  cccttgctcaccatggtaccACGACTCTCTCCAACTCCAAG  ccaaatcgactctagaATGGCCGAAGAATGCAGTGTT  cccttgctcaccatggtaccTCTATACAAACATCCTCTATATGTTggcgcgccactagtgATGGGGAGAAGCCCTTGTTGT  aaacgacggccagtgACGACTCTCTCCAACTCCAAGC ggcgcgccactagtggatccATGGCCGAAGAATGCAGTGT  gcggtaccctcgaggtcgacTCTATACAAACATCCTCTATATG |
